# Stochastic Control of Inertial Sea Wave Energy Converter

**DOI:** 10.1155/2015/980613

**Published:** 2015-03-22

**Authors:** Mattia Raffero, Michele Martini, Biagio Passione, Giuliana Mattiazzo, Ermanno Giorcelli, Giovanni Bracco

**Affiliations:** ^1^Department of Mechanics and Aerospace Engineering, Polytechnic University of Turin, Corso Duca degli Abruzzi 24, 10129 Turin, Italy; ^2^Environmental Hydraulics Institute “IH Cantabria”, C/Isabel Torres 15, Santander, 39011 Cantabria, Spain

## Abstract

The ISWEC (inertial sea wave energy converter) is presented, its control problems are stated, and an optimal control strategy is introduced. As the aim of the device is energy conversion, the mean absorbed power by ISWEC is calculated for a plane 2D irregular sea state. The response of the WEC (wave energy converter) is driven by the sea-surface elevation, which is modeled by a stationary and homogeneous zero mean Gaussian stochastic process. System equations are linearized thus simplifying the numerical model of the device. The resulting response is obtained as the output of the coupled mechanic-hydrodynamic model of the device. A stochastic suboptimal controller, derived from optimal control theory, is defined and applied to ISWEC. Results of this approach have been compared with the ones obtained with a linear spring-damper controller, highlighting the capability to obtain a higher value of mean extracted power despite higher power peaks.

## 1. Introduction

Oceans represent a wide, distributed reservoir of energy and, in them, waves are by far the most conspicuous form of energy. The global power potential represented by waves in open oceans, where energy is not dissipated due to seabed friction or wave breaking, has been estimated to be in the order of 10 TW (1 terawatt = 10^12^ W), a quantity comparable with the present world power demand [1].

For more than two centuries, many devices have been proposed for harvesting such a huge power source: the earliest patent was filed in 1799 in France [2]. Traditionally, the father of modern wave energy exploitation is considered Masuda, who started his studies in the 1940s and developed a navigation buoy powered by an air turbine which has been later commercialized [3]. Since then a lot of devices have been conceived and developed while a few of them arrived to the precommercial stage [4].

Among these machines, a considerable role is played by gyroscopic converters. Gyroscopes have been widely used on ships with the task of roll stabilization [5, 6]. The first use of gyroscopes for wave energy extraction is due to Salter, who invented the Duck device at the University of Edinburgh in the 1970s [7, 8]; the ISWEC belongs to the last generation of this kind of energy converters. Many problems have still to be solved in order to develop a reliable and economically sustainable wave energy converter (WEC). A proof of that is given by the little number of surviving WEC concepts [9, 10]. The main issue is the “reaction problem”: in order to generate an action on the power take-off (PTO: the component aimed at the power conversion, e.g., the electric generator) to generate energy, a reaction is needed and has to be given by either the seabed, the water, inertia, or other structures [11]. Moreover, sea waves involve low-frequency and alternating high forces, making it necessary to use strong structures and heavy conversion systems and therefore increasing the technology costs. Other problems to be faced are related to corrosion of components in contact with the sea water, possible leakage of oil (if hydraulic conversion systems are used), survivability in case of extreme events, maintenance, and environmental and visual impacts [12–15].

In this paper, one of the most important issues for the power optimization of a WEC is faced: the control problem. Developing a good control scheme is challenging and many solutions have been proposed in the recent years [16]. In Section 2, the main existing WECs control algorithms are described. The reviewed algorithms are as follows: the linear proportional-derivative (PD) controller, the latching and declutching controller, the optimal controller, and finally the stochastic suboptimal controller. Afterwards the ISWEC is presented and the equations describing its working principle are discussed. Given the physical characteristics of the full scale prototype, which will be installed in 2014 in real sea, a performance analysis is carried out, comparing the results obtained with the PD controller and the stochastic control algorithm, for some representative wave conditions registered at the installation site. Moreover, the effect of the maximum PTO torque constraint is analyzed in order to take into account the real machine limits.

## 2. WECs Control System Outlook

In this section, a review of the existing control algorithms for wave energy converters is given, so that the reader can have an overview of the state of the art in this field. In most cases, when analyzing the power extraction capabilities of a WEC, a one degree of freedom system is analyzed. As described in Section 3 of the paper, in the simplest case the hydrodynamic model of the device may be approximated by a 2nd order linear differential equation whose coefficients are frequency dependent. In the following considerations such a simple model may be a good reference for a reader that does not have a deep knowledge of this field.

Often, the first step is to develop a control strategy able to maximize the power output under plane (2D problem) monochromatic waves. Of course this means that the wave profile is composed of a single frequency contribution and this is not what happens in real sea. Afterwards, the case of plane polychromatic wave is analyzed generating a wave time series based on the spectrum of a specific sea state or using acquired wave data. In the most recent studies a 3D sea state is analyzed taking into account wave contributions coming from different directions.

Many control strategies have been proposed with varying levels of complexity. The main ones are described here.

### 2.1. PD Controller

One of the simplest ways to control a WEC is to apply on the floater an action proportional to its velocity. This kind of controller can be called “proportional controller (P)” and the ratio of force to velocity is the damping coefficient. In this case, the power output is related to the square of the wave height; moreover, if the wave is monochromatic and its frequency matches the natural frequency of the device, the velocity and the force are in phase and the power absorbed by the WEC is maximum [16]. The natural frequency of a floating body is dependent on its physical features and could be varied acting on its mass, for example, in order to match the incident wave frequency, thus maximizing its response amplitude. Another way to obtain such a result, without acting on the physical quantities of the device, is to use a reactive controller. This kind of controller can also be called “proportional-derivative controller (PD)” since the torque acting on the floater is composed of two contributions: the first one, proportional to the speed such as in the P controller, and the second one, proportional to the displacement of the body (with respect to the hydrostatic equilibrium condition). The ratio between the last force term and the displacement is the stiffness coefficient. As shown in Section 4.1 in this case it is possible to tune the response of the device in order to make the device resonant with the incoming wave [17]. A problem often arises with this kind of controller: the PTO can provide an action up to a maximum value, thus limiting the capacity of the system to adapt itself to the incoming wave. Moreover, the PD controller implies reactive power thus increasing the power losses due to the action generated by the PTO on the floater. After these considerations, it is clear that the floater has to be designed properly in order to reduce the control reactive component for most of the incoming waves.

### 2.2. Latching/Declutching Controller

The latching control technique has been firstly proposed for a heaving body, independently, by Falnes and Budal [18], French [19], and Guenther et al. [20]. This strategy is particularly suitable for waves longer than the WEC natural period; it basically consists in locking the floating body when its velocity approaches the zero value, by means of a clamping mechanism, and then releasing it at some point so that its velocity will be at its highest point simultaneously with the wave force; at this point the PTO force is set to its maximum value. The action on the system can thus be regarded as binary; that is, either the body is locked, or it is moving under maximum PTO action—thus resulting in a highly nonlinear control force. The declutching controller is similar to the previous one, but it is applied for waves shorter than the WEC natural period [21]. Different from before, the floater is normally free to move and when its velocity reaches some desired value, the maximum PTO force is applied.

The use of genetic algorithms indicated that, if applicable, the latching and declutching control is among the best control techniques for a wave energy converter; see Nolan et al. [22]. A drawback of these strategies is that they need some kind of prediction of the incoming wave force, in order to actuate the device at the right time (autoregressive models and Kalman filter have been widely used in this context); however, as an advantage with respect to the previously mentioned “PD controller,” any reactive power flow is eliminated from the power take-off. The result is a suboptimal control strategy that is best suitable using hydraulic power take-off systems. Experimental tests have been carried out during time, including wave prediction, which proved the reasonable goodness of these control strategies especially if compared to applying linear damping; see Budal et al. [23], Hals et al. [24], Falnes and Bjarte-Larsson [25], and Lopes et al. [26].

However, these considerations apply to devices for which the control force is directly applied on the floater main degree of freedom, so that this could be locked or released at the desired time instant. The wave energy converter considered in this paper is not suitable for the implementation of this strategy, since in such a device it is not possible to lock/release the relative motion between floater and gyro at a desired time instant.

### 2.3. Optimal Controller

Optimal control theory, as described in [27, 28], has already been applied on a wave energy converter model by Nielsen et al. [29]. The objective of this control strategy is to maximize the power transfer from waves to the floater in a wide range of sea states.

Here, the idea is to make the controller compensate for the dynamics of the floater and then damp its oscillation, so that its motion is in phase with the wave excitation force and thus the power flow is unidirectional, from the waves to the WEC. In this controller, an infinite time horizon is needed thus resulting in a noncausal control law. In order to overcome such noncausality, an approximation is introduced. The convolution integral is split into two parts: the causal part remains as it is, whilst the noncausal part is replaced by a damping term, whose value is obtained by means of a stochastic analysis of the wave-structure interaction aimed at maximizing the expected value of the power output. A more detailed explanation of this approach can be found in Section 4.3 of this paper after the hydrodynamic model description.

## 3. The ISWEC

In this section the ISWEC device is introduced. After a brief description of the device, the hydrodynamic model of the floater and the mechanical model of the gyroscope are described. Finally, the features of the ISWEC first full scale prototype, analyzed in this paper and to be deployed in autumn 2014, are reported.

### 3.1. Description of the System

ISWEC (inertial sea wave energy converter) is a device designed to exploit wave energy through the gyroscopic effect of a flywheel [30–33]. A lot of studies and experimental tests have been carried out on this device proving the concept feasibility [34, 35] and estimating its annual energy production [36].

**Figure 1:**
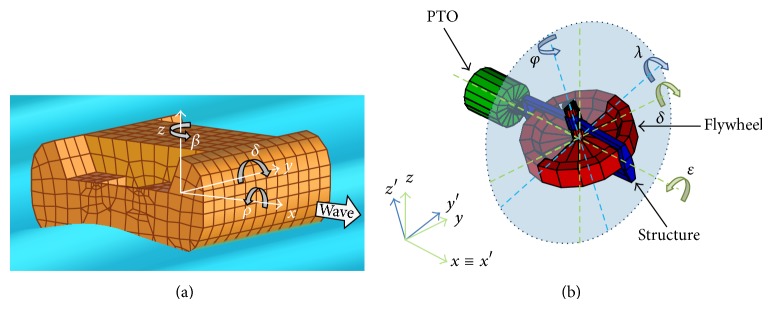
ISWEC geometry and coordinate systems.

Figure 1 shows the four main components of the gyroscopic system: the floater, the flywheel, the gyro structure, and the PTO. To describe the system dynamics, two reference frames have to be introduced: a hull-fixed coordinate system *x*,  *y*,  *z* and a gyroscope structure-fixed coordinate system *x*′, *y*′, *z*′. Both have their origins coincident with the centre of gravity of the system. The *x*-axis is oriented towards the bow and coincides with the sea wave direction. The hull rotates about the *y*-axis with the induced pitching motion *δ* due to the wave-floater-gyro interaction. Due to the angular momentum conservation of the flywheel, the combination of the pitch speed $\dot{\delta }$ with the flywheel speed $\dot{\varphi }$ about the *z*′-axis generates a gyroscopic torque *T*_*ε*_ around the *x*′-axis, which can be exploited by the PTO to generate electrical power. The device involves two main phenomena: the hull hydrodynamics and gyroscope mechanics. There is a strong coupling between them due to torques and energy interactions as shown in the following paragraphs.

The main advantages of the ISWEC device with respect to its competitors are the following ones. All the mechanical components of the system are enclosed in a sealed hull retained by a slack mooring line and, seen from outside, the system thus looks like a moored boat. This means that direct interaction between water and moving parts is avoided, thus reducing corrosion problems and maintenance. Moreover, the flywheel speed is an additional free parameter that can be tuned to increase the device performance in a wide range of wave conditions.

### 3.2. Dynamics of the Gyroscope

From the time derivation of the flywheel angular momentum, the equilibrium of the system is described in the gyro-frame coordinate system as in [30, 37]

(1) $$\begin{array}{c} T_{\varepsilon }=I\ddot{\varepsilon }+\left(I-J\right){\dot{\delta }}^{2}\sin\varepsilon \cos\varepsilon -J\dot{\varphi }\dot{\delta }\cos\varepsilon , \end{array}$$

(2) $$\begin{array}{c} T_{\varphi }=J\left(\ddot{\delta }\sin\varepsilon +\dot{\varepsilon }\dot{\delta }\cos\varepsilon +\ddot{\varphi }\right), \end{array}$$

(3) $$\begin{array}{c} T_{\lambda }=I\ddot{\delta }\cos\varepsilon +\left(J-2I\right)\dot{\varepsilon }\dot{\delta }\sin\varepsilon +J\dot{\varphi }\dot{\varepsilon }, \end{array}$$

where *I* represents the inertia of the gyroscopic system with respect to the *x*′- and *y*′-axes and *J* with respect to the *z*′-axis. The three torques are given to the gyroscopic system, respectively, by the PTO (1), the flywheel motor (2), and the hull (3). The torques given by the latter two equations have a key role in the system behavior: their projection on the *y*- and *z*-axes represents the pitch and yaw torques that the gyroscopic system discharges to the floater. In particular, for the torque related to the pitching *y*-axis, it is possible to write

(4) Tδ=Jsin2⁡ε+Icos⁡2⁡εδ¨ +Jφ¨sin⁡ε+Jφ˙ε˙cos⁡⁡ε+2J−Iδ˙ε˙sin⁡εcos⁡⁡ε.

Linearizing the mean zero position of the PTO shaft and assuming that the pitching accelerations of the system are small, respectively, from (1), (2), and (4), one gets eventually

(5) $$\begin{array}{c} T_{\varepsilon }=I\ddot{\varepsilon }-J\dot{\varphi }\dot{\delta }, \end{array}$$

(6) $$\begin{array}{c} T_{\varphi }=J\dot{\varepsilon }\dot{\delta }, \end{array}$$

(7) $$\begin{array}{c} T_{\delta }=J\dot{\varphi }\dot{\varepsilon }. \end{array}$$

These simple equations are very useful for a preliminary design of the hull, the gyroscope, the PTO, and the control system logic to be implemented on the machine [31]. These equations are supposed to be valid for small angles of oscillation; for the purpose of this study, results are considered valid for PTO oscillations amplitudes up to 45 degrees. The strong coupling between the floater and the gyroscope can be shown here. The action torque $J\dot{\varphi }\dot{\delta }$ given by the gyroscope to the PTO is function of the pitch speed. The result of such torque (combined with the control torque *T*_*ε*_) is the acceleration of the PTO shaft $\ddot{\varepsilon }$. The reaction torque $J\dot{\varphi }\dot{\varepsilon }$ given by the gyroscope to the floater is function of the PTO speed $\dot{\varepsilon }$ and, as shown in the next paragraph, interacts with the floater dynamics thus affecting the pitch motion $\dot{\delta }$.

### 3.3. Hydrodynamic Model and Full System Equation

#### 3.3.1. Cummins' Equation

For the pitch motion of a rigid floating marine structure, with zero forward speed, assuming that coupling with the other degrees of freedom is negligible, the equation of motion in the time domain can be written in body-fixed coordinates as

(8) $$\begin{array}{c} \left(I_{F}+A_{\infty }\right)\ddot{\delta }+\overset{t}{\underset{0}{\int }}h_{r\dot{\delta }}\left(t-\tau \right)\dot{\delta }\left(\tau \right)d\tau +K\delta =T_{w}-T_{m}-T_{\delta } \end{array}$$

according to Cummins' decomposition (1962), which studied the hydrodynamic problem under the assumption of linear phenomena [38]. This equation is valid only for small pitch oscillations; in the present study, oscillations up to 10 degrees in amplitude are considered physically meaningful. In the expression above, *δ* represents the pitch angle, *I*_*F*_ the floater inertia moment, *A*_*∞*_ the added mass for infinite oscillation frequency, and *K* the constant hydrostatic restoring force due to buoyancy and gravity, and finally $h_{r\dot{\delta }}$ is the impulse response function of the radiation forces. The convolution term models the radiation hydrodynamic problem in an ideal fluid with a linear pressure force distribution and it is often referred to as “fluid memory effect.”

The terms on the right hand side of the equation represent the pitch torque due to the incoming wave *T*_*w*_, the pitch torque due to the mooring forces *T*_*m*_, and the pitch control torque *T*_*δ*_ acting on the floater. Note that, as anticipated in Section 2.2, the control torque *T*_*δ*_ is generally directly given by the PTO, while in the ISWEC it is given by the gyroscope as a reaction torque due to its motion $\dot{\varepsilon }$  (7). Mooring contribution will be neglected here, under the assumption that its effect on the pitching motion of the device is small.

Ogilvie converted Cummins' equation for a free-floating body in the frequency domain, under only wave excitation forces, and found out the following relationships [39]:

(9) $$\begin{array}{c} \left[-\omega^{2}\left(I_{F}+A\left(\omega \right)\right)+j\omega B\left(\omega \right)+K\right]\cdot \delta_{0}=h_{w}\cdot f_{w}\left(\omega \right), \end{array}$$

(10) $$\begin{array}{c} H_{r}\left(j\omega \right)=B\left(\omega \right)+j\omega \left[A\left(\omega \right)-A_{\infty }\right], \end{array}$$

where *A* and *B* are, respectively, the frequency-dependent added mass and potential damping, while *H*_*r*_ is the frequency response function of the radiation. On the right hand side of the equation the wave excitation torque *T*_*w*_ is given by the frequency-dependent force coefficient *f*_*w*_, representing the torque per wave amplitude unit, multiplied by the wave amplitude *h*_*w*_ evaluated at the center of gravity of the floater. Note that relation (9) is written in the frequency domain thus involving linear quantities and steady state conditions; moreover such relation is valid under monochromatic excitation force. However relation (10) that describes the frequency response function of the radiation is very useful because it will be used in the next section for the implementation of the time domain model.

#### 3.3.2. Modeling of the Radiation Forces

The numerical computation of the convolution term in (8) may be quite time-consuming and not well suited for the design and analysis of the wave energy converter control system. Pérez and Fossen suggested a smart way for overcoming this problem [40]. Based on (10), it is possible to pursue a parametric frequency domain identification of the impulse response function. The objective is to find an appropriate order transfer function which satisfies the criteria of minimum approximation error, stability, and passivity. The frequency-dependent added mass *A* and potential damping *B* can be found for a chosen set of frequencies by means of any commercial code based on the implementation of the panel method under the assumption of potential flow. By means of the toolbox developed by Perez and Fossen it has been possible to identify the transfer function related to the pitching degree of freedom of the structure under investigation [41].

**Figure 2:**
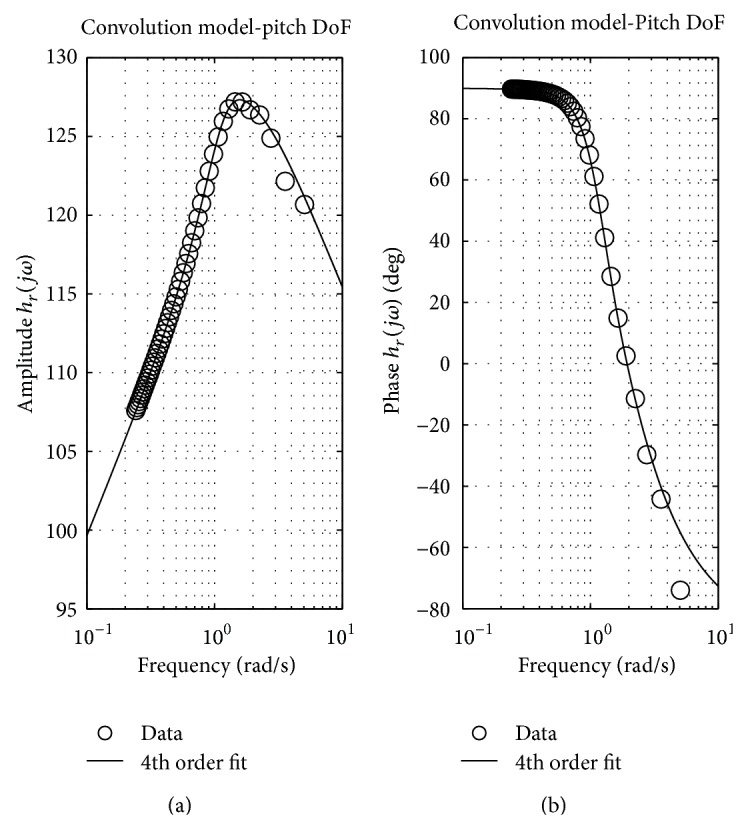
Frequency domain identification for the ISWEC floater: 4th order transfer function magnitude and phase.

Figure 2 shows that it has been possible to find a transfer function able to describe the radiation frequency response function of the floater, with a sufficient approximation in the typical frequency range of the studied sea. The obtained transfer function is stable and responds to the required passivity criteria.

#### 3.3.3. Modeling of the Wave Excitation Forces

With the assumption that the wave elevation process is a homogeneous and stationary zero-mean Gaussian process, the sea state is given by the one-sided wave spectrum *S*_ηη_^*^(*ω*). Given the RAO (response amplitude operator) of the system, *H*_*eη*_(*ω*), that describes the amplitude and phase of the force acting on the floater with respect to a unit amplitude monochromatic wave, it is possible to calculate the time history of the wave forces acting on the structure as a finite sum of harmonic excitation forces:

(11) $$\begin{array}{c} T_{w}\left(t\right)=\overset{M}{\underset{m=1}{\sum }}\overset{-}{T_{m}}\cos\left(\omega_{m}t+\phi_{m}+\theta_{m}\right), \end{array}$$

where

(12) $$\begin{array}{c} \overset{-}{T_{m}}=\sqrt{2{\left|H_{e\eta }\left(\omega_{m}\right)\right|}^{2}S_{\eta \eta }^{*}\left(\omega_{m}\right)\Delta \omega }\\ \phi_{m}=∠\left[H_{e\eta }\left(\omega_{m}\right)\right]. \end{array}$$

The angle *θ*_*m*_ between the harmonics components of the spectrum can either be chosen as random phase or can be guided by a groupiness factor [42] or, in case of wave data acquisitions, may be the phase angle given by the fast Fourier transform (FFT) analysis of the time series. The approach above described is referred to as linear stochastic wave load model [43].

### 3.4. Features of the Tested ISWEC Device

**Figure 3:**
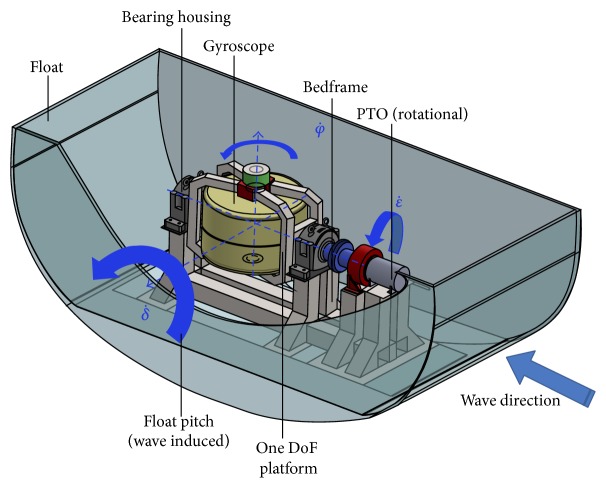
ISWEC layout concept.

The first full scale prototype of the ISWEC device will be installed in 2014 off Pantelleria Island (Sicily, Italy) [36°50′00′′N, 11°55′39′′E] (see Figure 3 and Table 1).

For the Pantelleria site a wave gauge has measured the sea wave elevation for the whole 2010. Among the acquired data, a set of nine 20-minute-long waves has been chosen as representative of the site as shown in Table 2.

The reported data are the result of a spectral analysis of the acquired time series where *H*_*m*0_ is the wave spectral height and *T*_*e*_ is the wave energy period.

## 4. Optimal Control of a Pitching Wave Energy Converter

In this section the control problem of a generic pitching device is introduced. Starting from the floater hydrodynamic equation, the maximum extractable power is obtained for both monochromatic and irregular wave. The suboptimal causal control algorithm is then introduced and the optimal damping factor is obtained by means of a stochastic analysis of the wave resource. In the next section the causal suboptimal control is applied on the ISWEC and its performances are compared with the linear reactive control.

### 4.1. Optimal Control under Monochromatic Wave

As stated in the Introduction, the PTO control force for the linear reactive controller is composed of two parts: an elastic contribution and a damping one. It can be written as follows:

(13) $$\begin{array}{c} T_{\delta }=-k\delta -b\dot{\delta }. \end{array}$$

Adding (13) in (9), the dynamic equation of the controlled system, in the frequency domain, eventually becomes

(14) $$\begin{array}{c} \left[-\omega^{2}\left(I_{F}+A\right)+j\omega \left(B+b\right)+\left(K+k\right)\right]\cdot \delta_{0}=h_{w}\cdot f_{w}. \end{array}$$

Given the incident wave frequency *ω*, the maximum power output is achieved by setting the proper *b*, *k* parameters that can be obtained applying the maximum power transfer theorem (Jacobi's Theorem, 1840):

(15) $$\begin{array}{c} b=B,\\ k=\left(I_{F}+A\right)\omega^{2}-K. \end{array}$$

In such conditions, the system is resonant with the incoming wave, so the force and the speed are in phase and the power extracted by the oscillator is

(16) $$\begin{array}{c} P_{m}=\frac{1}{8}\frac{{\left|f_{w}\right|}^{2}}{B}h_{w}^{2}. \end{array}$$

This result has been obtained with regular monochromatic wave, to show how it is possible to maximize the power extraction by tuning the control parameters. In case of irregular waves, the optimal parameters for the linear reactive controller can be found using an optimization algorithm [44].

### 4.2. Optimal Control of a Pitching Wave Energy Converter

An analytical approach may be followed to find an optimal control force law, which ensures the floating device to absorb the maximum mechanical energy from a given irregular sea state. This is usually called a deterministic optimal control problem and can be solved following basically two paths: the Hamilton-Jacobi-Bellman method [27] and Pontryagin's principle [28], based on a variational approach. In this analysis the latter method has been used, which is widely discussed and explained in [29, 45]. Given (8) that describes the dynamic of the floater and assuming that all the state variables are deterministic quantities, the control force that maximizes the mean absorbed power is found to be

(17) Tδ,optt=−IF+A∞δ¨t−Kδt +∫−∞+∞hrδ˙t−τδ˙τdτ.

As it can be noted such a control law is noncausal, depending on the future values of the velocity $\dot{\delta }$. Inserting the equation above into the equation of motion of the system (8), one can get the following:

(18) $$\begin{array}{c} \overset{\infty }{\underset{-\infty }{\int }}h_{r\dot{\delta }}\left(\left|t-\tau \right|\right)\dot{\delta }\left(\tau \right)d\tau =T_{w}\left(t\right). \end{array}$$

Equation (18) is also known to be a Fredholm integral equation. Fourier transforming it, one gets the following relationship between the wave excitation force and the pitching velocity at a general excitation frequency *ω* for optimal control:

(19) $$\begin{array}{c} T_{w}\left(\omega \right)=2B\left(\omega \right)\dot{\delta }\left(\omega \right), \end{array}$$

where *B*(*ω*) represents the potential damping of the system. Therefore, the optimal control law has as a direct consequence the fact that the wave excitation force is in phase with the floater pitching velocity for all their harmonic components, which is consistent with the hypothesis of maximum power transfer to the system.

### 4.3. Suboptimal Causal Feedback Control of a Pitching Wave Energy Converter

As previously stated, the control law proposed in the previous paragraph is noncausal and cannot be implemented on a real machine, unless the future evolution of the system is known or predicted with a sufficient level of accuracy. At this point, two possible approaches may be followed.

- The noncausal control law is used together with some prediction algorithm of the future incident wave force, as stochastic autoregressive models [46], neural networks [47], or digital filters.
- The optimal control law is approximated by a closely related causal process and the algorithm becomes then suboptimal. This method does not need to know the wave elevation in order to be used.

In this analysis the second approach will be followed, since the quality of the prediction algorithms is not considered high enough to control the ISWEC with the desired accuracy. The anticausal part of the convolution term in (17) is replaced as

(20) $$\begin{array}{c} \overset{+\infty }{\underset{t}{\int }}h_{r\dot{\delta }}\left(t-\tau \right)\dot{\delta }\left(\tau \right)d\tau ⟶2b_{c}\dot{\delta }\left(t\right). \end{array}$$

The new, causal-control force becomes then

(21) Tδ,optt=−IF+A∞δ¨t−Kδt +2bcδ˙(t)−∫−∞thrδ˙t−τδ˙τdτ.

The reason for using this control force is evident when replacing it by the equation of motion of system (8), where it follows that

(22) $$\begin{array}{c} T_{w}\left(t\right)=2b_{c}\dot{\delta }\left(t\right). \end{array}$$

This equation is similar to that one obtained for the noncausal optimal controller, with the difference that the damping coefficient is constant with respect to the frequency. Its value has to be determined by means of some optimality criterion for the mean absorbed mechanical power under given sea state conditions. For linear stiffness and monochromatic waves, it is easy to find that [29]

(23) $$\begin{array}{c} b_{c}=B\left(\omega \right). \end{array}$$

Instead, for the case of irregular waves, the calculation of the damping factor *b*_*c*_ can be related to a stochastic dynamic response analysis of the wave energy converter.

It can be argued that the impulse response function of the causal optimal stochastic controller is different, everywhere in the time domain, from that of the optimal stochastic controller. However, as pointed out and demonstrated in [29], “*the causal controller absorbs almost as much power as the optimal controller for all parameter values defining the autospectral density function,*” which gives confidence and robustness to the investigated methodology.

### 4.4. Stochastic Identification of the Damping Factor

Assuming that the floating device is in stationary conditions and keeping the assumption that the wave elevation can be regarded as a stationary zero-mean Gaussian process, through linear stochastic dynamics theory [45, 48], it is possible to derive the optimal control law for known sea state conditions. Under these assumptions, the pitching velocity process and in turn the displacement and acceleration can be regarded as stationary zero-mean Gaussian, independent random processes. Moreover due to stationary conditions the following properties hold [45, 49]:

(24) $$\begin{array}{c} E\left[\dot{\delta }\left(t\right)\right]=0, \end{array}$$

(25) $$\begin{array}{c} E\left[\delta \left(t\right)\dot{\delta }\left(t\right)\right]=0⟶E\left[\dot{\delta }\left(t\right)\ddot{\delta }\left(t\right)\right]=0, \end{array}$$

(26) $$\begin{array}{c} E\left[\dot{\delta }\left(t\right)\dot{\delta }\left(t\right)\right]=\sigma_{\dot{\delta }}^{2}, \end{array}$$

(27) Eδ˙tδ˙t+Δt=κδ˙δ˙Δt⟶Eδ˙tδ˙τ=κδ˙δ˙τ−t=κδ˙δ˙t−τ,

where the operator *E*[·] indicates the expected value and *κ* the autocorrelation function that in case of zero-mean process is equal to the covariance function. The substitution

(28) $$\begin{array}{c} \Delta t=\tau -t \end{array}$$

has been applied in order to match the notation used in the Cummins equation (8). For the suboptimal control and assuming that all responses processes are ergodic, the mean absorbed power becomes

(29) Pa−=ETδtδ˙t=−IF+A∞Eδ˙tδ¨t+2bcEδ2˙t −KEδ˙(t)δt−∫−∞thrδ˙t−τEδ˙tδ˙τdτ.

Using the relations from (24) to (27) the equation above becomes

(30) Pa−=2bcσδ˙2−∫−∞thrδ˙t−τκδ˙δ˙t−τdτ=2bcσδ˙2−∫0∞hrδ˙uκδ˙δ˙udu.

By means of the Wiener-Khinchin theorem which relates the Fourier transform of the autocorrelation function of a stationary random process to its double-sided autospectral density function, we get the following:

(31) κδ˙δ˙τ=∫−∞∞eiωτSδ˙δ˙ωdω=∫−∞∞eiωτSFeFeω4bc2dω=κFeFeτ4bc2.

Given the sea state, the spectrum of the wave excitation forces can be obtained, with it being related to the spectrum of the wave elevation process through the wave-to-force response amplitude operator as

(32) $$\begin{array}{c} S_{F_{e}F_{e}}\left(\omega \right)={\left|H_{e\eta }\left(\omega \right)\right|}^{2}S_{\eta \eta }\left(\omega \right). \end{array}$$

Moreover, the variance of the velocity process is related to that of the wave excitation force process by

(33) $$\begin{array}{c} \sigma_{\dot{\delta }}^{2}=\overset{\infty }{\underset{-\infty }{\int }}S_{\dot{\delta }\dot{\delta }}\left(\omega \right)d\omega =\overset{\infty }{\underset{-\infty }{\int }}\frac{S_{F_{e}F_{e}}\left(\omega \right)}{4b_{c}^{2}}d\omega =\frac{\sigma_{F_{e}}^{2}}{4b_{c}^{2}}. \end{array}$$

The mean absorbed power in (30) becomes then

(34) Pa−=2bcσδ˙2−∫0∞hrδ˙uκδ˙δ˙udu=σFe212bc−14bc2∫0∞hrδ˙uρFeFeudu,

where *ρ*_*F*_*e*_*F*_*e*__ is the autocorrelation coefficient function of the wave excitation force process and is defined as

(35) $$\begin{array}{c} \rho_{F_{e}F_{e}}\left(\tau \right)=\frac{\kappa_{F_{e}F_{e}}\left(\tau \right)}{\sigma_{F_{e}}^{2}}. \end{array}$$

The maximum for the absorbed power function (34) is then finally found for a damping value of

(36) $$\begin{array}{c} b_{c}=\overset{\infty }{\underset{0}{\int }}h_{r\dot{\delta }}\left(u\right)\rho_{F_{e}F_{e}}\left(u\right)du. \end{array}$$

Therefore, once the sea state and the hull hydrodynamic properties are known, it is possible to calculate the suboptimal, unconstrained, stochastic value of the damping coefficient for the pitching motion of the system.

## 5. Results: Control Strategy for the ISWEC

The previously mentioned control law was obtained and tested for a generic pitching device (acting directly on the floater by means of a control torque *T*_*δ*_); in this section, it will be used to control the ISWEC gyroscope in order to maximize the wave power conversion. The ISWEC can be controlled acting through the PTO on the *ε*-axis of the gyroscope by means of the control torque *T*_*ε*_. Two main control strategies are tested for the device under consideration, with and without PTO torque saturation, and the results are compared.

### 5.1. Proportional Derivative (PD) Control Law for ISWEC

A simple and easy controller consists in making the PTO behave as a spring-damper group. This approach was proposed being similar to the one presented in “PD Controller.” The PTO torque equation can be written as

(37) $$\begin{array}{c} T_{\varepsilon }=-k_{\varepsilon }\varepsilon -c_{\varepsilon }\dot{\varepsilon }. \end{array}$$

The goal is now to maximize the PTO mean power production on a wide range of sea conditions. For the chosen set of waves, the best stiffness and damping values in terms of mean power production were calculated by means of a parametric analysis.

**Figure 4:**
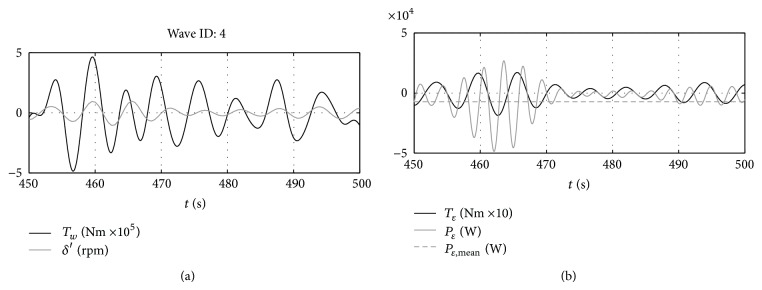
Time histories of the main system variables. (a) Wave excitation force versus pitch speed and (b) PTO torque and power.

From Figure 4, it can be noticed that the power flux between the PTO and the gyroscope is bidirectional (i.e., the PTO sometimes acts as a motor), therefore introducing a reactive power component. This is why this kind of control is also referred to as “reactive control.”

**Figure 5:**
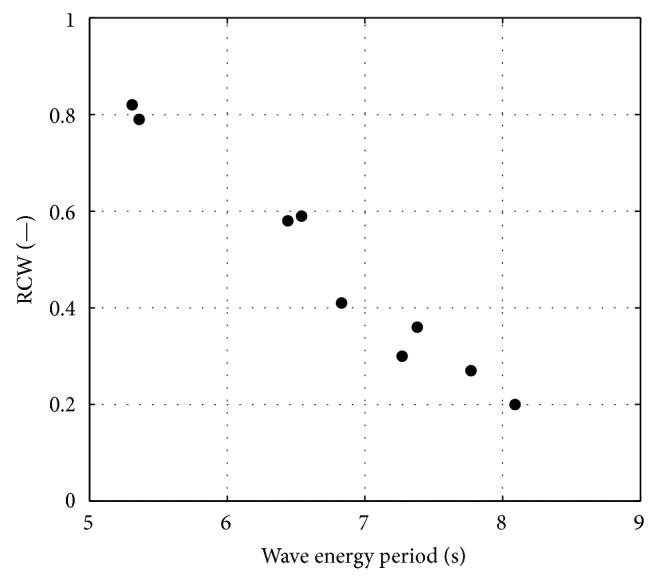
RCW for the optimized linear reactive controller.

The goodness of the conversion efficiency, in order to provide a further comparison parameter for the same device using different control laws, is related here to the relative capture width, RCW, calculated as

(38) $$\begin{array}{c} \text{RCW}=\frac{\overset{-}{P_{\varepsilon }}}{P_{\text{wave front}}}=\frac{\overset{-}{\left(T_{\varepsilon }\dot{\varepsilon }\right)}}{0.49H_{m0}T_{e}^{2}W}, \end{array}$$

where *W* is the floater width. This term is the ratio between the mean mechanical power generated by the device (which is assumed to be equal to the electrical power, i.e., electrical conversion efficiency equal to unity) and the power of the wave front and it represents somehow the transfer of energy from the wave to the floating device; it should be noted however that its value may exceed one, since the floater may absorb more energy than the one contained in the wave front due to wave-body interactions [50, 51]. An interesting trend is found if this indicator is plotted versus the wave energy period (Figure 5).

Since the ISWEC pitching undamped natural period is approximately 5.5 s, the waves with longer period are less suitable for power extraction with this kind of device [34].

### 5.2. Suboptimal Stochastic Control of ISWEC

The suboptimal causal control law calculated in the previous section has been implemented on the ISWEC device. The objective is to control the gyroscope to ensure that a given torque *T*_*δ*_ is discharged to the hull. Once the optimal pitching torque is calculated through (21) and (36), the PTO speed to be set for the linear gyroscope is calculated by means of (7) as

(39) $$\begin{array}{c} {\dot{\varepsilon }}_{\text{set}}=\frac{T_{\delta ,\text{opt}}}{J\dot{\varphi }}=\frac{T_{\delta ,\text{opt}}}{L}, \end{array}$$

where *L* is the angular momentum of the flywheel. A closed-loop speed control is implemented acting on the PTO torque. Moreover, since the gyroscopic torque acting as a disturbance on the PTO axis is known analytically from (5), it is possible to add a feedforward torque as

(40) $$\begin{array}{c} T_{\varepsilon ,ff}=-J\dot{\varphi }\dot{\delta }=-L\dot{\delta }. \end{array}$$

The gyroscopic torque makes the PTO speed deviate from its target value, and the feedforward torque is used to cancel out this effect and help the control to work better.

**Figure 6:**
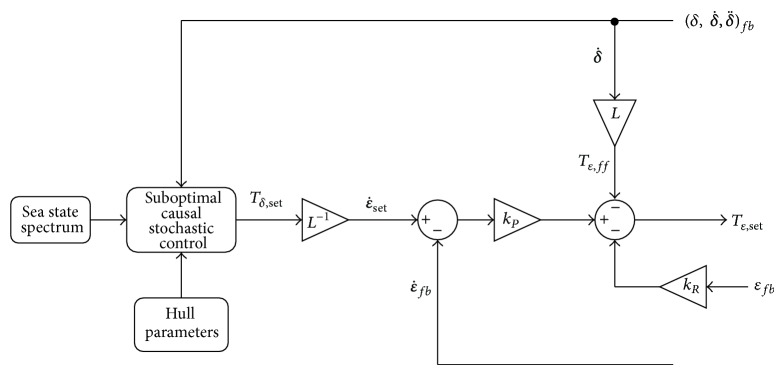
Closed loop optimal speed control for the ISWEC.

The resulting control system for the ISWEC device is shown in Figure 6.

*k*_*P*_ is the proportional gain of the closed-loop speed controller and it has been necessary to introduce a relatively small stiffness *k*_*R*_ term in order to prevent position drifting in irregular wave conditions. Notice that the hull parameters are known since they are characteristics of the device, while the sea state spectrum is given by the weather forecast and by an on-board monitoring system that will be installed for the sea state evaluation and prediction.

### 5.3. Unconstrained Optimal Control

**Figure 7:**
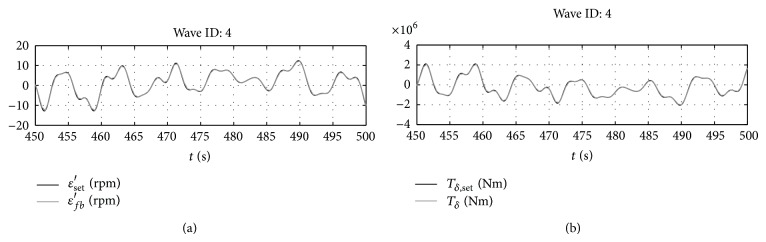
Set versus feedback for gyroscope speed and pitching torque.

**Figure 8:**
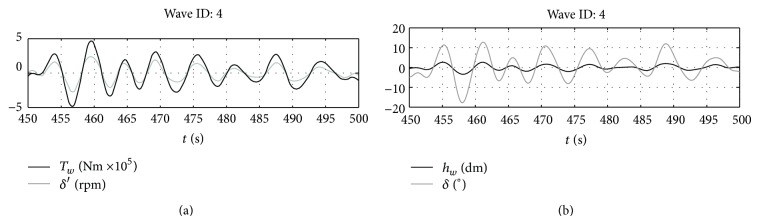
(a) Wave force versus pitching velocity and (b) wave elevation versus pitch angle.

It is initially considered that the PTO can give any torque to the shaft. In these conditions, the behavior of the system is represented in Figures 7 and 8 for the representative wave number 4.

As it can be seen in Figure 7, with the implemented control loop, the gyroscope is able to produce the pitching torque required by optimal control by rotating at the required speed. As already seen for the monochromatic wave in Section 4.1, when the optimal control is implemented, the wave excitation torque and the pitching velocity of the floating device are “in phase”; that is, their maxima occur at the same time instants (Figure 8). This is one of the first consequences of the implementation of the optimal control law.

It is interesting that correspondingly the pitching position of the device is reasonably in phase with the wave measured at the body centre of gravity. This may be very useful in further development of the control algorithm of the system. At the same time, the oscillations of the gyroscope are relatively small which ensures some grade of reliability in using the linearized gyroscope equations. The same holds for the pitching oscillations of the device. Results for the other waves are summarized in Table 4.

In this section, the results for the waves numbers 2, 5, 6, 7, and 8 were excluded due to high pitching floater oscillations, for which the linear hydrodynamic model loses its validity. Compared with Table 3, it can be noticed that the RCW of the optimal controlled system is higher but higher peak torque values are registered too.

### 5.4. Unconstrained Optimal Control with Constraints

In a real machine, the PTO undergoes some current and thus torque limitations. In order to be able to apply the methodology shown before, it would be needed to recalculate optimal control signals with respect to system constraints, for example, torque limitations. This could be the object of future investigations; nonetheless, it is interesting to show the effect of imposing system constraints a posteriori to the optimal unconstrained control signals. Two different values for the PTO maximum torque have been investigated and the results are reported in Table 5. When saturations occur, the gyroscope is no longer able to control the floater motion as requested by the optimal control algorithm. This is reflected in the fact that the wave force and the pitching velocity lose their phasing during this transition. As shown in Table 5 for 500 kNm PTO saturation torque, the overall effect is a decrease in the mean power extracted by the machine and thus in the RCW.

**Figure 9:**
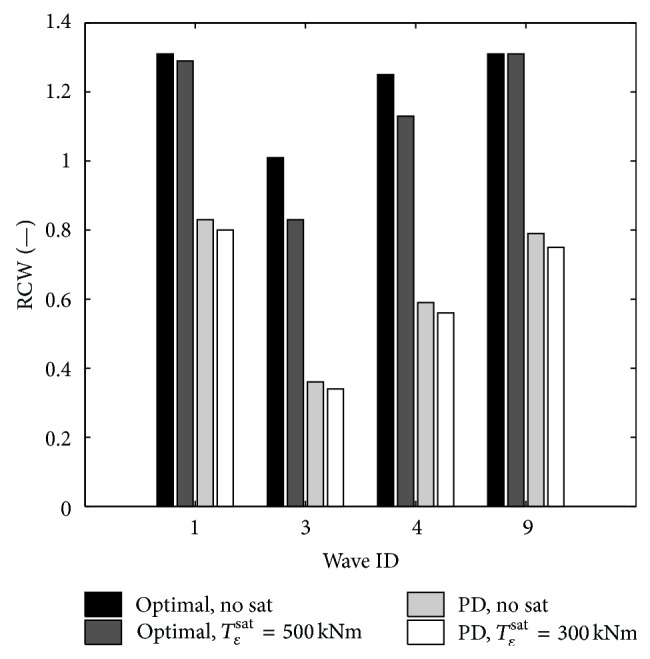
ISWEC RCW comparison between optimal and linear reactive controller with and without torque saturation.

It has not been possible to decrease more the PTO saturation torque in this section since the oscillations of the gyroscope were too high for the linear model to be still acceptable (Figure 9).

An interesting result is found: since the maximum torque for the linear reactive controller was about 500 kNm, the same value has been imposed on the constrained optimal control and though the power production decreases with respect to the optimal unconstrained case, it is higher than the one extracted with the linear reactive controller.

## 6. Conclusions

Stochastic suboptimal control and linear reactive control have been developed, tested, and compared for the ISWEC device. Results were obtained with a linear dynamic model of the system. The suboptimal control maximizes the mean absorbed power at the cost of higher power peaks and generator torques if compared with an optimized linear reactive controller applied to the gyroscope. However if torque limitation is imposed, the power production is still higher than the one obtained with the linear reactive controller. Nonetheless, the optimal control theory can give an upper bound of the performance of the WEC under irregular sea state conditions and furnishes guidelines for the optimization of other control algorithms and its parameters can be derived analytically given the sea state and the hull hydrodynamic properties. Further studies are needed in order to assess the nonlinear gyroscope performance and controllability. Moreover, the hydrodynamic model has to be improved in order to take into account nonlinear wave forces and wave-body interactions when high pitch angles are involved.

Energy dissipations have to be introduced in the model in order to maximize the net power production of the system. Comparison with experimental data will be carried out once the ISWEC prototype is installed and tested in real sea conditions.

## Figures and Tables

**Table 1:** Features of the full scale ISWEC prototype.

Property	Value	Units
Hull width	8	m
Hull length	15	m
Hull natural period	5.5	s
Gyro mass	20	ton
Gyro diameter	3	m

**Table 2:** Spectral properties of the chosen set of waves.

Wave ID	Wave spectral height	Wave energy period	Wave power density
[—]	[m]	[s]	[kW/m]
1	1.18	5.31	3.65
2	1.97	6.44	12.25
3	0.67	7.38	1.61
4	0.68	6.54	1.50
5	1.36	6.83	6.23
6	2.20	8.09	19.18
7	1.45	7.77	8.06
8	1.99	7.27	14.16
9	0.69	5.36	1.25

**Table 3:** Results obtained for the linear reactive controller: main physical variables that characterize the system response to the considered waves.

Wave ID	*c*_*ε*_	*k*_*ε*_	*δ*_max⁡_	*T*_*δ*,max⁡_	*ε*_max⁡_	*T*_*ε*,max⁡_	*P*_*ε*,max_	*P*_*ε*,mean_	*P*_*ε*,max⁡_/*P*_*ε*,mean_	RCW
[]	[kNm s]	[kNm]	[°]	[MNm]	[°]	[kNm]	[kW]	[kW]	[]	[]
1	1036	−5	13.9	0.712	20.3	469	0	24	0.0	0.83
2	537	746	35.7	2.002	66.7	1079	277	81	3.4	0.81
3	104	278	7.5	1.026	41.7	203	49	5	10.5	0.36
4	144	455	10.8	1.054	37.6	319	76	7	10.4	0.59
5	537	455	15.8	1.026	34.6	434	33	21	1.6	0.42
6	278	278	16.2	1.580	66.0	422	77	33	2.3	0.21
7	104	278	13.7	1.722	65.7	371	143	19	7.5	0.29
8	200	278	16.2	1.808	66.6	421	111	40	2.8	0.35
9	746	455	7.7	0.485	15.1	250	5	8	0.6	0.79

**Table 4:** Results for the optimal stochastic control with no constraints.

Wave ID	*δ*_max⁡_	*T*_*δ*,max⁡_	*ε*_max⁡_	*T*_*ε*,max⁡_	*P*_*ε*,max_	*P*_*ε*,mean_	*P*_*ε*,max⁡_/*P*_*ε*,mean_	RCW
[]	[°]	[MNm]	[°]	[kNm]	[kW]	[kW]	[]	[]
1	28.4	3.47	22.0	822	805	39	20.5	1.31
3	30.6	5.90	26.0	897	1448	13	110.7	1.01
4	29.5	4.05	21.8	813	836	15	54.8	1.25
9	16.7	1.65	14.2	576	238	14	17.6	1.31

**Table 5:** Results with saturation on PTO torque at 500 kNm.

Wave ID	*δ*_max⁡_	*T*_*δ*,max⁡_	*ε*_max⁡_	*T*_*ε*,max⁡_	*P*_*ε*,max_	*P*_*ε*,mean_	*P*_*ε*,max⁡_/*P*_*ε*,mean_	RCW
[]	[°]	[MNm]	[°]	[kNm]	[kW]	[kW]	[]	[]
1	19.5	6.02	55.3	500	1430	38.2	37.5	1.29
3	27.7	7.53	63.0	500	1340	10.5	127.9	0.83
4	26.1	6.48	68.8	500	1350	13.5	99.7	1.13
9	15.6	1.66	14.9	500	237	13.5	17.6	1.31

## Nomenclature

*A*: Added mass of the floater in the frequency domain
*A*_*∞*_: Added mass for infinite oscillation frequency
*B*: Hydrodynamic damping coefficient in the frequency domain
*E*[]: Expected value of
*F*_*e*_: Wave excitation force in the frequency domain
*H*_*eη*_: Force-to-motion response amplitude operator of the system
*H*_*m*0_: Wave spectral height
*H*_*r*_: Frequency response function of the radiation
*I*: Inertia moment of the gyroscopic system with respect to the *x*′- and *y*′-axes
*I*_*F*_: Inertia moment of the floater with respect to the *y*-axis
*J*: Inertia moment of the gyroscopic system with respect to the *z*′-axis
*K*: Hydrostatic restoring force (hydrostatic stiffness)
*L*: Angular momentum of the flywheel
${\overset{-}{P}}_{a}$: Absorbed mean power under stochastic optimal causal control
*P*_*m*_: Mean power extracted by the oscillator under optimal control
*P*_wave front_: Input power from the wave front
${\overset{-}{P}}_{\varepsilon }$: Mean extracted power by the PTO
RCW: Relative capture width of the WEC
*S*_*ii*_: Double-sided spectrum of the variable
*S*_*ηη*_^*^: One-sided wave spectrum
*T*_*δ*_: Floater control torque/gyro reaction torque on *y*-axis (given to the hull)
*T*_*δ*,opt_: Optimal floater control force
*T*_*e*_: Wave energy period
*T*_*m*_: Mooring reaction torque
$\overset{-}{T_{m}}$: Amplitude of the *m*th harmonic of the wave excitation force
*T*_*w*_: Wave excitation torque
*T*_*ε*_: Torque on the *x*′-axis (from the PTO to the gyroscopic system)
*T*_*λ*_: Torque on the *y*′-axis (from the hull to the gyroscopic system)
*T*_*φ*_: Torque on the *z*′-axis (from the flywheel motor to the gyroscopic system)
*W*: Floater width
*b*: Damping coefficient of the optimal control
*b*_*c*_: Damping coefficient of the optimal causal control
*c*_*ε*_: Damping coefficient of the PD control
*f*_*w*_: Froude-Krylov forces coefficient
$h_{r\dot{\delta }}$: Impulse response function of the radiation forces with respect to the pitching motion
*h*_*w*_: Wave amplitude
*k*: Stiffness coefficient of the optimal control
*k*_*ε*_: Stiffness coefficient of the PD control
*t*: Time
*δ*: Pitch angle (rotation about the *y*-axis)
*ε*: PTO angle (rotation about the *x*′-axis)
*θ*_*m*_: Angle between the harmonics components of the wave spectrum
*κ*_*xx*_: Autocorrelation function
*σ*^2^: Variance
*ϕ*_*m*_: Phase angle of *H*_*eη*_
$\dot{\varphi }$: Flywheel angular velocity (rotation about the *z*′-axis)
*ω*: Angular frequency.
